# Intraplant and Interspecific Antioxidant Interactions in *Origanum vulgare* and *Mentha aquatica*

**DOI:** 10.3390/molecules31071110

**Published:** 2026-03-27

**Authors:** Elena Kurin, Svetlana Dokupilová, Lucia Račková, Pavel Mučaji, Silvia Bittner Fialová

**Affiliations:** 1 Department of Pharmacognosy and Botany, Faculty of Pharmacy, Comenius University Bratislava, Odbojárov 10, 83232 Bratislava, Slovakia; 2 Department of Pharmaceutical Analysis and Nuclear Pharmacy, Faculty of Pharmacy, Comenius University Bratislava, Odbojárov 10, 83232 Bratislava, Slovakia; 3 Institute of Chemistry, Slovak Academy of Sciences, Dúbravská Cesta 9, 84538 Bratislava, Slovakia; 4 Centre of Experimental Medicine, Institute of Experimental Pharmacology and Toxicology, Slovak Academy of Sciences, Dúbravská Cesta 9, 84104 Bratislava, Slovakia; 5 Department of Pharmaceutical Technology, Pharmacognosy and Botany, University of Veterinary Medicine and Pharmacy in Košice, Komenského 73, 04181 Košice, Slovakia

**Keywords:** *Origanum vulgare*, *Mentha aquatica*, antioxidant activity, phytochemical analysis, plants mixtures

## Abstract

The antioxidant activity of *Origanum vulgare* L. and *Mentha aquatica* L. has been widely reported; however, interaction effects within and between different plant parts remain insufficiently characterized. This study aimed to evaluate the antioxidant behavior of methanolic extracts from leaves, flowers, and rhizomes of both species and to assess the nature of intraplant and interspecific interactions using combination analysis. Antioxidant activity was determined for individual extracts and their binary mixtures using DPPH and ABTS radical scavenging assays. Phytochemical analysis was performed by LC-MS/MS. In *O. vulgare*, all intraplant mixtures exhibited synergistic effects, suggesting complementary contributions of phenolic acids and flavonoids across plant organs. In contrast, *M. aquatica* showed more variable responses, with additive to antagonistic interactions, particularly in combinations involving rhizomes with lower phenolic content. Interspecific mixtures further demonstrated that interaction outcomes depended on the qualitative and quantitative composition of phytochemicals: leaf mixtures showed synergism, whereas flower and rhizomes mixtures tended toward antagonism. Comparable interaction trends were observed in both radical scavenging assays. These results indicate that antioxidant activity in plant mixtures is not simply additive but is strongly influenced by phytochemical composition and plant part, highlighting the importance of empirical testing when designing multicomponent plant-based antioxidant formulations.

## 1. Introduction

Water mint (*Mentha aquatica* L.) and oregano (*Origanum vulgare* L.) are perennial members of the Lamiaceae family with considerable industrial relevance. The aerial parts of these species are widely utilized, not only in traditional medicine but also as valuable raw materials in the food and cosmetic industries.

*M. aquatica* L. is rich in essential oil, which comprises monoterpenes (e.g., menthofuran, menthol, menthone, limonene) and sesquiterpenes (e.g., viridiflorol, ledol, β-caryophyllene) [1]. The plant is rich in phenolic acids, including rosmarinic acid, caffeic acid, and chlorogenic acid, and contains flavonoids such as eriocitrin, luteolin-glucoside, isorhoifolin, kaempferol 3-*O*-glucoside, and hesperidin, previously identified by HPLC analyses [2,3]. Recent studies highlight its pharmacological potential, particularly antioxidant, antimicrobial, and neuroactive properties [1]. Several in vitro studies have demonstrated significant radical-scavenging and cytoprotective effects of water mint extracts. The biological effects of water mint have been confirmed not only individually but also in combination. Importantly, a synergistic effect was observed when *M. aquatica* extract was combined with *Lavandula dentata*, resulting in enhanced cytoprotective activity, likely mediated by rosmarinic acid and flavanones. These findings highlight the relevance of water mint not only as a potent natural antioxidant but also as a promising component of synergistic phytochemical formulations [4].

*Origanum vulgare* L., a perennial species of the Lamiaceae family, is native to the Mediterranean, Euro-Siberian, and Irano-Turanian regions. Its aerial parts, particularly the leaves, are widely utilized as a culinary spice due to their essential oil content, which imparts a characteristic aroma and flavor. The chemical profile of oregano essential oil is highly variable and depends on environmental conditions, with major constituents including thymol, carvacrol, γ-terpinene, p-cymene, β-caryophyllene, limonene, ocimene, linalool, and 4-terpinenol [5,6]. Beyond essential oils, oregano is also a rich source of polyphenols, mainly flavonoids such as luteolin, apigenin, and quercetin derivatives, as well as phenolic acids, particularly rosmarinic acid [7]. Oregano exhibits diverse biological activities, including antioxidant, anti-inflammatory, antimicrobial, and antiviral effects [8]. Traditionally, it has been applied for self-medication of digestive disturbances, respiratory infections, urinary tract diseases, and oral health problems [9]. In the food industry, oregano is valued not only as a flavoring agent but also as a natural preservative, contributing to product quality through its antioxidant potential and its ability to limit microbial growth [10,11]. Interest in natural antioxidants has increased as the food industry seeks safer and more sustainable alternatives to synthetic preservatives. Plant-derived extracts, including oregano, are therefore being explored for their potential to enhance food quality and stability across various matrices. Studies in meat systems have shown that oregano-derived compounds can contribute to improved oxidative stability and quality retention during storage [12,13]. In addition, oregano phytochemicals have been successfully applied in edible coatings, helping delay spoilage processes and limit microbial growth in fresh produce [14]. These findings highlight oregano as a multifunctional plant source whose antioxidant properties are not only biologically relevant but also technologically valuable, justifying further investigation of its antioxidant interactions and efficacy.

Despite the well-documented antioxidant properties of *O. vulgare* or *M. aquatica*, the interaction patterns arising from their combined use remain insufficiently understood. While most studies focus on single extracts or isolated compounds, accumulating evidence suggests that the biological activity of plant-based preparations is largely governed by complex phytochemical interactions rather than by individual constituents alone. Synergistic, additive, or antagonistic effects may emerge depending on the qualitative and quantitative composition of metabolites, their molecular structures, and relative concentrations. Therefore, a systematic evaluation of intra- and interspecific combinations of plant parts represents a crucial step toward understanding how phytochemical diversity shapes antioxidant efficacy. In this context, the present study aims to elucidate the interaction behavior of methanolic extracts from different organs of *O. vulgare* and *M. aquatica*, with particular emphasis on identifying synergistic mixtures and exploring the mechanistic basis of their antioxidant interactions.

## 2. Results

The phenolic profiles of *Origanum vulgare* L. and *Mentha aquatica* L. were characterized by LC–MS/MS. Analysis revealed the presence of phenolic acids, flavonoids, and their glycosylated derivatives distributed differentially among rhizomes, leaves, and flowers. Compounds were identified by comparing their retention times, molecular ions [M–H]^−^, and MS/MS fragmentation patterns with literature data and available standards (Table 1).

In *O. vulgare*, a total of thirteen phenolic compounds were detected, including flavonoids (apigenin, luteolin, chrysoeriol derivatives) and phenolic acids (caffeic acid, rosmarinic acid, salvianolic acid B, and oreganol derivatives). Several compounds, such as apigenin-7-*O*-glucuronide and luteolin-7-*O*-glucuronide, were identified based on their characteristic fragmentation patterns, typically involving neutral losses of glucuronic acid (176 Da) or hexose moieties (162 Da). Protocatechuic acid glycosides (oregano A and C), as well as caffeic acid derivatives, were identified by comparing with our previous work [15]. Chrysoeriol-7-O-(6″-*O*-acetyl)-allosyl-(1→2)-glucoside and its diacetylated form were tentatively identified by their high molecular masses (*m*/*z* 665 and 707, respectively) and characteristic MS/MS fragment ions resulting from successive losses of sugar and acetyl groups. Chrysoeriol-C-hexoside-C-pentoside, detected in rhizomes, showed a typical C-glycosyl flavonoid fragmentation pattern, which agrees with literature data describing such compounds in Lamiaceae species [16,17]. Among phenolic acids, rosmarinic acid was the most abundant compound in methanolic extracts of *O. vulgare* different organs with particularly high concentrations in leaves (245.6 ± 3.5 µg/mg dry extract) and flowers (248.0 ± 5.3 µg/mg dry extract). This confirms rosmarinic acid as the major phenolic constituent of oregano, as widely reported. Oreganol A and oreganol C, both protocatechuic acid esters, were also present and mainly accumulated in aerial parts, whereas salvianolic acid B was detected only in rhizomes. The organ-specific distribution suggests differential biosynthesis or translocation of phenolics within the plant, with rhizomes acting as reservoirs for more complex phenolic acids.

In *M. aquatica*, nine phenolic compounds were identified, with flavonoid glycosides and rosmarinic acid being dominant. Eriodictyol and its glycosides (eriodictyol-7-*O*-glucoside and eriodictyol-7-neohesperoside) were identified based on the product ion at *m*/*z* 287, corresponding to the eriodictyol aglycone. Similarly, diosmin and hesperidin were identified by their diagnostic fragment ions at *m*/*z* 299 and 301, respectively, reflecting typical flavanone glycoside fragmentation. All identified phenolics have been reported across the genus *Mentha* in the past [3,18]. Rosmarinic acid was again the quantitatively dominant compound in *M. aquatica*, reaching the highest concentration in flowers (153.0 ± 5.5 µg/mg dry extract), followed by leaves (116.3 ± 7.4 µg/mg dry extract) and rhizomes (157.5 ± 1.7 µg/mg dry extract).

Overall, both species exhibited a clear dominance of rosmarinic acid and its derivatives, highlighting the importance of the phenylpropanoid pathway in Lamiaceae. However, *O. vulgare* was richer in complex phenolic acid esters (oreganols and salvianolic acid B), whereas *M. aquatica* showed a higher diversity of flavanone and flavone glycosides. The distinct qualitative and quantitative profiles among plant organs indicate that phenolic metabolism is strongly organ-dependent, with aerial parts being more varied and richer in phenolic substances. Nevertheless, rhizomes are also a significant source of biologically active secondary metabolites.

The total polyphenol content of methanolic extracts from different plant parts of *Mentha aquatica* and *Origanum vulgare* was determined to assess their phenolic profiles (Table 2). Extracts were prepared by macerating dried plant material in methanol, followed by filtration and adjustment to final volumes. The resulting dry extract from 1 g plant material are present in Table 2 and correspond to total polyphenol contents of these extracts. Notably, the extracts from leaves generally exhibited higher polyphenol levels compared to rhizomes and flowers, with O. vulgare leaves showing the highest content. The values reported in Table 2 represent the measured phenolic content expressed as µg gallic acid equivalents per mL of extract (µg GAE/mL).

**Figure 1:**
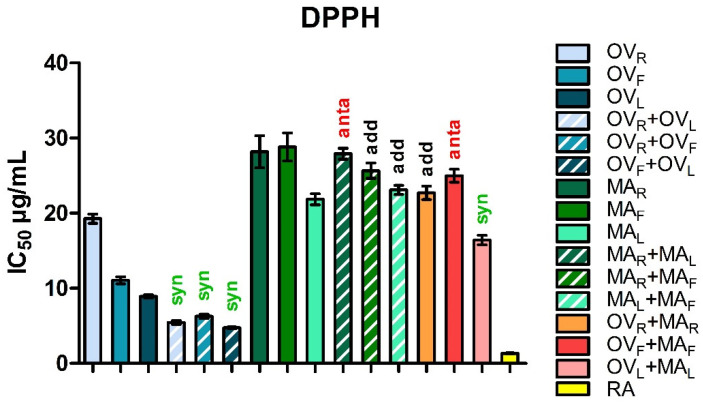
DPPH radical scavenging activity (expressed as IC_50_, µg/mL) of *Origanum vulgare* (OV) and *Mentha aquatica* (MA) methanolic extracts and their binary combinations. Bars represent mean ± SE, n = 4. Rosmarinic acid (RA) served as a positive control. Interaction types are indicated above bars: syn—synergism (CI < 1; green), add—additive effect (CI ≈ 1; black), anta—antagonism (CI > 1; red). Abbreviations: OV_R_, OV_F_, and OV_L_ refer to *O. vulgare* rhizome, flower, and leaf extracts, respectively; MA_R_, MA_F_, and MA_L_ correspond to *M. aquatica* rhizome, flower, and leaf extracts.

**Figure 2:**
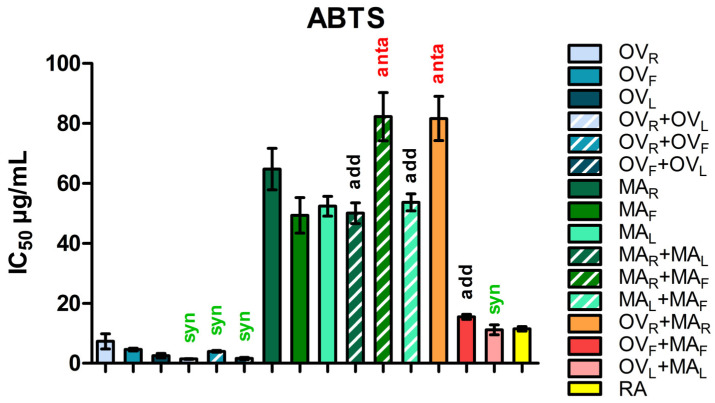
ABTS radical scavenging activity (expressed as IC_50_, µg/mL) of *Origanum vulgare* (OV) and *Mentha aquatica* (MA) methanolic extracts and their binary combinations. Bars represent mean ± SE, n = 4. Rosmarinic acid (RA) served as a positive control. Interaction types are indicated above bars: syn—synergism (CI < 1; green), add—additive effect (CI ≈ 1; black), anta—antagonism (CI > 1; red). Abbreviations: OV_R_, OV_F_, and OV_L_ refer to *O. vulgare* rhizome, flower, and leaf extracts, respectively; MA_R_, MA_F_, and MA_L_ correspond to *M. aquatica* rhizome, flower, and leaf extracts. Prior to testing, methanolic extracts of each plant part were prepared at concentrations corresponding to the amount of dry residue obtained from 1 g of starting plant material. Binary mixtures were then formed by combining equal volumes of the individual stock solutions, resulting in variable ratios of extract solids depending on the extraction yields of each part. These ratios are reported in brackets (Table 3) throughout the results to indicate the relative contribution of each extract in the mixture. This approach enabled the interaction analysis to be performed on independently prepared extracts of individual plant organs, allowing the contribution of each extract to the mixture to be defined based on its extraction yield.

The antioxidant capacity of methanolic extracts from *Origanum vulgare* (OV) and *Mentha aquatica* (MA), as well as their binary mixtures, was evaluated using the DPPH and ABTS radical scavenging assay. The results are summarized in Table 2 and Table 3 and visualized in Figure 1 and Figure 2. Among the individual plant parts, *O. vulgare* leaves (OV_L_) exhibited the highest activity (DPPH: IC_50_ = 8.92 ± 0.20 µg/mL; ABTS: IC_50_ = 2.54 ± 0.72 µg/mL), followed by flowers (OV_F_; DPPH: IC_50_ = 11.04 ± 0.46 µg/mL; ABTS: IC_50_ = 4.60 ± 0.47 µg/mL) and rhizomes (OV_R_; DPPH: IC_50_ = 19.27 ± 0.62 µg/mL; ABTS: IC_50_ = 7.34 ± 2.57 µg/mL). In *M. aquatica*, antioxidant activity was considerably lower, with IC_50_ values for rhizomes (MA_R_; DPPH: IC_50_ = 28.18 ± 2.14 µg/mL; ABTS: IC_50_ = 64.80 ± 6.90 µg/mL) and flowers (MA_F_; DPPH: IC_50_ = 28.81 ± 1.86 µg/mL; ABTS: IC_50_ = 49.37 ± 5.91 µg/mL), while leaves (MA_L_; DPPH: IC_50_ = 21.84 ± 0.73 µg/mL; IC_50_ = 52.44 ± 3.28 µg/mL) showed slightly higher activity than the other M. aquatica extracts in the DPPH assay but not in the ABTS assay. Rosmarinic acid (RA), used as a positive control, showed the strongest antioxidant activity (IC_50_ = 11.56 ± 0.77 µg/mL).

Binary intraplant combinations of *O. vulgare* demonstrated consistent synergism, with combination index (CI) values ranging from 0.48 to 0.53 in the DPPH assay and 0.47 to 0.78 in the ABTS assay. The CI parameter indicates the nature of the interaction, where CI < 1 denotes synergism, CI = 1 indicates additivity, and CI > 1 suggests antagonism. Among the tested combinations, OV_F_ + OV_L_ exhibited one of the strongest synergistic effects (DPPH: CI = 0.48 ± 0.01; IC_50_ = 4.76 ± 0.11 µg/mL; ABTS: CI = 0.50 ± 0.02; IC_50_ = 1.61 ± 0.38 µg/mL), indicating a markedly enhanced radical scavenging potential compared to the individual extracts. In the DPPH assay, strong synergistic interactions were observed for OV_R_ + OV_F_ (CI = 0.50 ± 0.01), while OV_R_ + OV_L_ also exhibited synergy, albeit to a slightly lesser extent (CI = 0.53 ± 0.01). Conversely, in the ABTS assay, the trend was reversed: OV_R_ + OV_L_ showed the strongest synergy (CI = 0.47 ± 0.01), whereas OV_R_ + OV_F_ displayed a weaker synergistic effect (CI = 0.78 ± 0.03).

These combinations also exhibited high dose-reduction indices (DRI up to 20.02), showing that substantially lower amounts of both extracts were sufficient to achieve 50% inhibition compared with the individual components. The DRI expresses how much the dose of each extract can be reduced in a synergistic mixture without loss of efficacy.

In *M. aquatica*, intraplant mixtures showed mostly additive or weakly antagonistic behavior in the DPPH assay. The MA_R_ + MA_L_ combination displayed slight antagonism (CI = 1.19 ± 0.01), while MA_R_ + MA_F_ and MA_L_ + MA_F_ were nearly additive (CI = 0.90–1.01). In the ABTS assay, the interactions differed: MA_R_ + MA_L_ was nearly additive (CI = 0.90 ± 0.03), MA_R_ + MA_F_ showed antagonism (CI = 1.40 ± 0.05), and MA_L_ + MA_F_ remained close to additive (CI = 1.04 ± 0.03). Dose reduction index (DRI) values for all combinations ranged from 1.18 to 5.25, indicating the potential for reduced extract doses while maintaining efficacy.

Interspecific mixtures between *O. vulgare* and *M. aquatica* parts showed a broader range of interactions in the DPPH assay. The leaf mixture (OV_L_ + MA_L_) demonstrated slight synergism (CI = 0.87 ± 0.01; IC_50_ = 16.41 ± 0.64 µg/mL; DRI up to 5.3). In contrast, the flower combination (OV_F_ + MA_F_) produced slight antagonism (CI = 1.15 ± 0.02). The rhizome mixture (OV_R_ + MA_R_) behaved nearly additively (CI = 0.97 ± 0.01). The ABTS assay showed a different pattern: OV_L_ + MA_L_ displayed stronger synergy (CI = 0.67 ± 0.02; IC_50_ = 11.19 ± 1.67 µg/mL; DRI up to 5.25), OV_F_ + MA_F_ was nearly additive (CI = 0.95 ± 0.03), and OV_R_ + MA_R_ exhibited pronounced antagonism (CI = 5.59 ± 0.32).

Overall, the data indicate that synergistic effects were most pronounced in *O. vulgare* intraplant combinations, particularly in leaves and flowers, as well as in leaf-based interspecific mixtures with *M. aquatica*. In contrast, *M. aquatica* intraplant combinations and rhizome- or flower-based interspecific mixtures generally exhibited additive or weakly antagonistic interactions, with some cases of pronounced antagonism observed in the ABTS assay.

## 3. Discussion

The antioxidant activity of *Origanum vulgare* (OV) [11,19,20] and *Mentha aquatica* (MA) [1,3,21] has been extensively documented, yet the reported outcomes remain variable. Our findings confirm that the antioxidant behavior of methanolic extracts from both species is strongly shaped by the plant species, plant part, and the specific type of mixture tested. In particular, both intraplant mixtures (combinations of different parts within the same plant) and interspecific mixtures (combinations of the same parts between the two species) exhibited distinct interaction patterns, underscoring the crucial role of phytochemical composition in modulating antioxidant efficacy. The diversity of responses, ranging from synergistic to additive and antagonistic effects, illustrates the complexity of phytochemical interplay in multicomponent plant systems [22,23]. Such variability is expected, as numerous studies have shown that the biological effects of phytochemicals depend on their concentration, molecular structure, and interactions with co-occurring compounds, which together shape their bioavailability and overall activity [24].

In OV extracts, all intraplant combinations showed synergy, likely reflecting the complementary distribution of metabolites across organs. Leaves and flowers were rich in rosmarinic acid, a potent phenolic antioxidant [25,26], along with smaller amounts of luteolin 7-*O*-glucuronide. Leaves further accumulated phenolic glycosides such as oreganol A (4′-*O*-ß-D-glucopyranosyl-3′,4′-dihydroxybenzyl protocatechuate) and oregano C (4′-*O*-β-D-glucopyranosyl-4′-hydroxybenzyl protocatechuate), while flowers contained oreganol A only. These compounds exhibit radical-scavenging activity and protection against oxidative stress. Oreganol A likely exerts its activity via hydrogen and electron donation from hydroxyl groups, while glycosylation, though lowering intrinsic reactivity, enhances bioavailability and may thus improve overall efficacy [27]. Rhizomes contained roughly threefold less rosmarinic acid and fewer phenolics but contributed distinct compounds, including unique chrysoeriol C-hexoside-C-pentoside, increasing chemical diversity. The observed synergistic behavior across all OV intraplant mixtures was confirmed in both DPPH (CI = 0.48–0.53; DRI up to 13.8) and ABTS assays (CI = 0.47–0.78; DRI up to 20.02), indicating that synergy persists across different radical systems. The combination of phenolic acids, glycosides, and flavonoids creates a diverse antioxidant pool in which cooperative interactions among structurally distinct molecules enhance the overall capacity. The combination of phenolic acids, glycosides, and flavonoids creates a diverse antioxidant pool, within which synergy likely arises from cooperative interactions among structurally distinct molecules. These interactions may involve regeneration of stronger antioxidants by weaker ones, formation of stable intermolecular complexes, solubility-driven mechanisms, or other unpredictable interactions that collectively enhance the overall antioxidant capacity [28].

In contrast, *M. aquatica* displayed more variable intraplant interactions, ranging from additive to antagonistic. Leaves contained high levels of eriocitrin, eriodyctiol derivatives, and rosmarinic acid, representing a potent antioxidant reservoir [25,29,30], while flowers contained the same compounds but at lower concentrations, along with diosmin and hesperidin, which exhibit weak activity [29]. Rhizomes were phenolic-poor, containing mainly rosmarinic acid and traces of diosmin. Intraplant interactions were consistent across assays in trend but differed in magnitude: for DPPH, leaves–rhizomes showed antagonism (CI = 1.19 ± 0.01), while leaf–flower and flower–rhizome mixtures were nearly additive (CI = 1.01 ± 0.01 and 0.90 ± 0.01, respectively); for ABTS, leaf–rhizome mixtures were closer to additive (CI = 0.90 ± 0.03), leaf–flower mixtures remained near additive (CI = 1.04 ± 0.03) and flower–rhizome combinations became antagonistic (CI = 1.40 ± 0.05). The antagonism in leaf–rhizome DPPH mixtures likely results from the dilution of potent antioxidants by weaker components and the overall lower concentration of phenolic compounds, including diosmin, which contributes minimally to radical scavenging activity [29], while the shift in ABTS reflects differential radical-specific reactivity.

Leaf–flower and flower–rhizome mixtures were mainly additive or slightly antagonistic, consistent with limited chemical novelty between these parts.

Although our present focus was on intraplant combinations, these findings conceptually align with the broader interspecific interaction framework. Accumulating evidence indicates that the biological activity of plant-based preparations often arises from interactions within multi-plant mixtures rather than from isolated constituents, in accordance with the principles of traditional phytotherapy [31]. Interspecific mixtures similarly demonstrated radical-dependent effects: in DPPH, rhizome mixtures (OV_R_ + MA_R_) were nearly additive (CI = 0.97 ± 0.01), flower mixtures (OV_F_ + MA_F_) showed slight antagonism (CI = 1.15 ± 0.02), and leaf mixtures (OV_L_ + MA_L_) displayed slight synergism (CI = 0.87 ± 0.01; DRI up to 5.3); in ABTS, the trend was partially reversed, with OV_R_ + MA_R_ exhibiting strong antagonism (CI = 5.59 ± 0.32), OV_F_ + MA_F_ near additive (CI = 0.95 ± 0.03), and OV_L_ + MA_L_ showing stronger synergy (CI = 0.67 ± 0.02).

In this context, the interspecific combinations further supported the view that chemical composition dictates the nature of interactions. Previous work on *Mentha* rhizomes (*Mentha* × *piperita*, *M. longifolia*, and *M.* × *villosa*) revealed only additive effects in all mutual binary combinations in the DPPH assay [26]. Similarly, the interspecific rhizome mixture (OV_R_ + MA_R_; CI = 0.97 ± 0.01) exhibited additive behavior, likely constrained by the low phenolic content of both parts. Notably, *Mentha* × *piperita* and *M.* × *villosa* mixtures showed additive effects in DPPH but slight antagonism in ABTS, reflecting a trend consistent with our observed interspecific rhizome mixtures. In contrast, flower mixtures (OV_F_ + MA_F_; CI = 1.15 ± 0.02) displayed antagonism in the DPPH assay and additivity in the ABTS assay (CI = 0.95 ± 0.03), plausibly due to competition among abundant phenolics for radical targets. Gojak-Salimović and Ramić [32] similarly observed that mixtures of caffeic, ferulic, and rosmarinic acids shifted from synergistic to antagonistic behavior with increasing concentration, supporting the view that compound abundance and overlap strongly influence interaction outcomes.

Leaves of both species (OV_L_ + MA_L_; DPPH: CI = 0.87 ± 0.01; DRI up to 5.3; ABTS: CI = 0.67 ± 0.02; DRI up to 5.25) showed synergistic effect, attributable to shared richness in rosmarinic acid, lacked diosmin, and distinct flavonoid profiles: *M. aquatica* leaves contained eriocitrin and eriodyctiol derivatives (including neoeriocitrin), whereas *O. vulgare* leaves accumulated oreganol A and C and luteolin 7-*O*-glucuronide. A similar pattern of synergistic enhancement was observed in the flavanone mixture composed of neohesperidin, hesperidin, neoeriocitrin, eriocitrin, and hesperetin, which displayed markedly higher antioxidant activity than the individual flavanones [33]. Eriocitrin has also been reported to act synergistically with α-tocopherol, further illustrating its cooperative antioxidant behavior [34].

Overall, these results demonstrate that the magnitude and direction of antioxidant interactions depend on both the radical type and the specific plant combination, with synergistic effects prevalent in *O. vulgare* intraplant mixtures and leaf-based interspecific mixtures, while antagonism or additivity was more frequent in *M. aquatica* intraplant mixtures and flower- or rhizome-based interspecific mixtures. Synergistic effects may result from regeneration of antioxidants with more negative one-electron redox potential E^0^ by weaker ones, formation of stable intermolecular complexes, or complementary mechanisms involving radical scavenging. Conversely, antagonism may arise from reverse regeneration (in which weaker antioxidants consume stronger ones) or from differences in the case of membranous cellular systems that hinder their interaction [35]. This aligns with previous observations that plant-derived antioxidants do not act in isolation but rather interact dynamically, sometimes unpredictably. Further targeted studies using defined mixtures of isolated compounds are needed to unravel these hidden interaction mechanisms and confirm whether the observed synergistic or antagonistic outcomes stem from specific molecular reactions or from broader compositional dynamics. In the future, advances in chemoinformatics and machine learning may help improve the prediction of such interactions in complex phytochemical mixtures, although experimental validation will likely remain essential.

## 4. Materials and Methods

### 4.1. Plant Material

Dried whole plants of *Origanum vulgare* L. at the stage of full flowering were obtained from the Medicinal Plants Garden of the Faculty of Pharmacy (48°08′33.6″ N, 17°11′21.6″ E), Comenius University Bratislava. *Mentha aquatica* L. was collected from the wild nature, in the Modra area, Holombecká dolina reservoir (48°20′23.0″ N 17°17′43.4″ E) in September 2020, at the stage of full flowering. *M. aquatica* was collected from the wild due to the absence of this species in the cultivation collection of the Medicinal Plants Garden. Silvia Bittner Fialová, PhD, performed the botanical identification of the plant material. The plant material was ground to a fine powder before extraction. The powdered plant material (rhizomes, leaves, or flowers) was extracted with methanol (raw material-to-solvent ratio approximately 1:16, *w*/*v*) using ultrasound-assisted extraction for 30 min at laboratory temperature. The mixtures were filtered, and the filtrates were adjusted to a final volume of 25 mL (10 mL for the flower) with methanol. Methanol was selected as an extraction solvent due to its high efficiency in extracting a broad range of polar secondary metabolites and its common use in phytochemical screening studies. For the determination of extractive yield, 10 mL of each extract was evaporated to dryness using a rotary evaporator, and the residue was weighed gravimetrically. For each plant part, a single extract was prepared (one biological replicate). Subsequent analytical measurements were performed in triplicate, and antioxidant assays were carried out in four technical replicates to ensure measurement reproducibility.

### 4.2. Chemicals and Reagents

Methanol, acetonitrile, water, and acetic acid used for extraction and chromatographic analyses were of LC–MS grade and purchased from Merck (Darmstadt, Germany). The following analytical reference standards were used for compound identification and quantification: rosmarinic acid (≤96%, HPLC), caffeic acid (≥98.0%, HPLC), salvianolic acid B (≥94%, HPLC), luteolin (purity > 95%), hesperidin (≥97.0%, HPLC) and diosmin (≥90.0%, HPLC) were obtained from Sigma-Aldrich (St. Louis, MO, USA). Eriocitrin (85.89%) was obtained from HWI pharma services GmbH (Rülzheim, Germany). Luteolin-7-*O*-glucoside ≥ 95.0% (HPLC) and luteolin-7-*O*-glucuronide ≥ 90.0% (HPLC) were obtained as phyproof^®^ Reference Substances from PhytoLab GmbH & Co. KG (Vestenbergsgreuth, Germany). For antioxidant assays, 2,2-diphenyl-1-picrylhydrazyl (DPPH), 2,2′-azinobis(3-ethylbenzothiazoline-6-sulfonic acid) (ABTS), and potassium persulfate (K_2_S_2_O_8_) were purchased from Sigma-Aldrich (St. Louis, MO, USA). All reagents were used without further purification.

### 4.3. Phenolics Identification by LC–MS/MS

LC–MS/MS was performed using an Agilent 1260 Infinity LC system (Agilent Technologies, Santa Clara, CA, USA) coupled to a 6520 Accurate-Mass QTOF mass spectrometer (ESI, Agilent Technologies, Santa Clara, CA, USA) equipped with an electrospray ionization (ESI) source operating in negative ion mode. The ESI parameters were set as follows: capillary voltage of 3.5 kV, nebulizer pressure of 40 psi (N_2_), drying gas flow of 10 L·min^−1^, and drying gas temperature of 300 °C. Data were acquired in autoMS/MS mode over an *m*/*z* range of 100–3000, with collision energy set to 20 eV. Compounds identification was achieved by comparing MS/MS spectra with the MassBank database (Version: v2025.12.1), literature data, or their retention times, molecular ions [M–H]^−^, and MS/MS fragmentation patterns with available analytical reference standards. The analytical separation method (HPLC) is described below.

### 4.4. Phenolics Quantification by HPLC-DAD

HPLC analysis was carried out using a SYKAM system equipped with a KINESIS TELOS^®^ LU C18 (Kinesis Ltd., Cambridgeshire, UK) (250 × 4.6 mm, 5 µm) at 30 °C. The mobile phase consisted of water with acetic acid (pH 2.41, phase A) and acetonitrile (phase B), using a gradient elution consisting of 23% B (0 min), 33% B (10 min), 45% B (20 min), 95% (22 min), 23% (25 min) at a flow rate of 0.8 mL·min^−1^. Detection was performed at 280 nm for flavonoids and protocatechuic acid esters and at 330 nm for phenolic acids. Quantification was based on external calibration curves (5–100 ppm) prepared from luteolin (primary RS, purity > 95%, Sigma-Aldrich, St. Louis, MO, USA) and rosmarinic acid standards (≤96%; Sigma-Aldrich, St. Louis, MO, USA). All standards showed good linearity (r^2^ > 0.995). Limits of detection (LOD) and quantification (LOQ) were calculated for both standards, and a mass accuracy tolerance of 10 ppm was applied for compound identification.

### 4.5. Preparation of Plant Part Extracts

Aliquots of 1 mL of the methanol extract were transferred into Eppendorf tubes, one of which was evaporated to determine the dry extract mass, and the remaining filtrate was used as the stock solution for subsequent analyses. The concentrations of these stock solutions (mg/mL), indicated in brackets throughout the results (Table 3), were prepared to maintain equivalent volumes based on the extraction yields of each plant part. A stock solution of rosmarinic acid (≤96%; Sigma-Aldrich, St. Louis, MO, USA) was prepared at 1.1 mg/mL in methanol and serially diluted to obtain a concentration series used as a positive control in the DPPH assay.

### 4.6. Preparation of Binary Plant Part Mixtures

Binary mixtures of rhizome + leaf, rhizome + flower, and leaf + flower were prepared by combining equal volumes (250 µL each) of the respective stock solutions and adjusting with methanol to 1 mL. Each mixture was homogenized and serially diluted 1:1 with methanol to generate five concentrations for antioxidant assays.

### 4.7. DPPH Radical Scavenging Assay

The antiradical activity of the samples was evaluated using the DPPH radical scavenging assay, adapted from Blois [36] with minor modifications, namely that the assay was performed in 96-well microplates instead of cuvettes. The stable free radical 2,2-diphenyl-1-picrylhydrazyl (DPPH; Sigma-Aldrich, St. Louis, MO, USA) was freshly prepared in methanol at a concentration of 55 µM. For the assay, 225 µL of the DPPH solution was combined with 25 µL of each test sample, which included individual extracts and their mixtures dissolved in methanol, as well as rosmarinic acid dissolved in methanol. Following a 30-min incubation at room temperature, the absorbance was recorded at 517 nm using a Tecan Infinite M200 microplate reader (Tecan AG, Grödig/Salzburg, Austria) in 96-well microplates (Greiner Bio-One GmbH, Frickenhausen, Germany). The percentage of DPPH radical scavenging was calculated according to Equation (1):

(1) $$\%\;radical\;scavenging\;activity=100\times \frac{A_{DPPH}-A_{sample}}{A_{DPPH}}$$

where A_DPPH_ is the absorbance of the control (DPPH solution without sample) and A_sample_ is the absorbance in the presence of the extract or standard. All measurements were carried out in quadruplicate, and IC_50_ values were calculated from the resulting dose–response curves using median-effect analysis via CompuSyn software version 1.0.1 (ComboSyn, Inc., Paramus, NJ, USA).

### 4.8. ABTS Radical Scavenging Assay

The antiradical activity of the samples was evaluated using the ABTS radical scavenging assay, adapted from Re et al. [37]. In total, 7 mM aqueous solution of 2,2′-azinobis-(3-ethylbenzothiazoline-6-sulfonic acid) (ABTS) (Sigma-Aldrich, St. Louis, MO, USA) was freshly prepared and mixed in an equimolar ratio with 2.45 mM potassium persulfate (K_2_S_2_O_8_) (Sigma-Aldrich, St. Louis, MO, USA). The mixture was kept in the dark at room temperature for 24 h to allow the formation of ABTS radicals. The resulting ABTS solution was then diluted with ethanol (1.1 mL) to a final volume of 50 mL. A 2.5 µL amount of each test sample, which included individual extracts and their mixtures dissolved in methanol, and rosmarinic acid dissolved in methanol was added to 247.5 µL of the ABTS solution. Absorbance changes were recorded after 6 min at 734 nm using a Tecan Infinite M200 microplate reader (Tecan AG, Grödig/Salzburg, Austria) in 96-well microplates (Greiner Bio-One GmbH, Frickenhausen, Germany). The percentage of ABTS radical scavenging was calculated according to Equation (2):

(2) $$\%\;radical\;scavenging\;activity=100\times \frac{A_{ABTS}-A_{sample}}{A_{ABTS}}$$

where A_ABTS_ is the absorbance of the control (ABTS solution without sample) and A_sample_ is the absorbance in the presence of the extract or standard. All measurements were carried out in quadruplicate, and IC_50_ values were calculated from the resulting dose–response curves using median-effect analysis via CompuSyn software version 1.0.1 (ComboSyn, Inc., Paramus, NJ, USA).

### 4.9. Interaction Analysis/Synergy Evaluation

The interaction type between extracts expressed as synergistic, additive, or antagonistic was quantified using the Combination Index (CI) method originally formulated by Chou [38]. This approach is based on the median-effect principle, which mathematically describes the relationship between dose and effect in mixtures.

The CI for an n-component system producing x% inhibition was obtained using Equation (3):

(3) (CI)nx=∑j=1nDj/(Dx)j

where ^n^(CI)_x_ represents the sum of the doses of *n* drugs that exert x% inhibition in a mixture. In the denominator (D_x_), D “alone” inhibits the system by x%. If the CI value equals 1, the interaction is considered additive; values below 1 indicate synergism, whereas values above 1 reflect antagonism.

Sequential deletion analysis (SDA), an iterative sequential deletion of one dose of a drug at a time for repetitive CI calculations and *r*-value analysis, which represents the conformity parameter for goodness of fit to the median-effect principle of the mass-action law were calculated using a median-effect analysis.

The Dose Reduction Index (DRI) was also determined to estimate the magnitude by which the dose of each extract in a combination could be reduced while maintaining the same inhibitory effect, as described in Equation (4):

(4) $${\left(DRI\right)}_{1}=\frac{{(D}_{x}{)}_{1}}{{(D}_{1})};{\left(DRI\right)}_{2}=\frac{{(D}_{x}{)}_{2}}{{(D}_{2})};\ldots ;etc.$$

A DRI value greater than 1 indicates a favorable reduction in the required dose, where higher DRI values correspond to more substantial dose savings at the same efficacy level. However, elevated DRI values do not necessarily imply synergism.

The CI, SDA and DRI values were obtained by median-effect analysis using CompuSyn software (version 1.0.1; ComboSyn Inc., Paramus, NJ, USA).

### 4.10. Statistical Analysis

All measurements were performed in quadruplicate. Dose–effect relationships were analyzed according to the median-effect equation described by Chou [38], and IC_50_ values were calculated using CompuSyn software version 1.0.1 (ComboSyn, Inc., Paramus, NJ, USA). The correlation coefficient (*r*-value) was obtained as a measure of goodness of fit, with all *r* ≥ 0.96, confirming excellent agreement with the median-effect principle. The standard error (SE) of the IC_50_ value was calculated from the covariance matrix of the regression parameters using standard error propagation, according to the procedure described previously [25].

## 5. Conclusions

This study demonstrated that the antioxidant interactions of *Origanum vulgare* and *Mentha aquatica* methanolic extracts are highly dependent on the plant part and the specific combination tested, and the type of radical assay used. In *O. vulgare*, intraplant mixtures consistently showed synergy, reflecting the complementary roles of phenolic acids and flavonoids. In *M. aquatica*, however, interactions were more variable, with antagonism arising particularly in combinations involving rhizomes, and the magnitude of interactions differed between DPPH and ABTS. Interspecific mixtures further confirmed that synergistic or antagonistic outcomes cannot be predicted solely from the activity of individual plant parts but depend on the qualitative and quantitative composition of metabolites as well as the radical system tested. These findings emphasize the necessity of empirical testing when designing plant-based formulations and highlight the potential of chemically diverse mixtures, particularly leaf extracts, as promising candidates for robust synergistic antioxidant activity across multiple radical types.

## Figures and Tables

**Table 1:** Chemical composition of methanolic extracts of different organs (rhizomes, leaves, flowers) of *O. vulgare* and *M. aquatica*.

Compound		RT	[M–H]^−^	MS/MS	Rhizomes *	Leaves *	Flowers *
					(µg/mg Dry Extract)	(µg/mg Dry Extract)	(µg/mg Dry Extract)
*Origanum vulgare*	Apigenin 7-*O*-[6″-*O*-acetyl]-allosyl(1→2)glucoside	7.88	635	431/269	LOQ	ND	ND
	Apigenin-7-*O*-glucuronide	9.115	445	269	ND	2.3 ± 0.0	ND
	Caffeic acid **	6.1	179	135	LOQ	ND	ND
	Chryseriol 7-*O*-[6″-O-acetyl]-allosyl(1→2)glucosid	5.95	665	623/299	LOQ	ND	ND
	Chrysoeriol 7-O-[6″-*O*-acetyl]-allosyl-(1→2)-[6″-*O*-acetyl]-glucosid	15.6	707	665/647/503/299	LOQ	ND	ND
	Chrysoeriol-acetyl-pentosyl-glucoside	12.55	635	593/299	LOQ	ND	ND
	Chrysoeriol-C-hexoside-C-pentoside	10.29	593	299	49.6 ± 2.2	ND	ND
	Luteolin 7-*O*-allosyl-(1→2)-[6″-*O*-acetyl]-glucoside	7.54	651	591/447/285	LOQ	ND	ND
	Luteolin-7-*O*-glucuronide **	5.918	461	285	ND	29.9 ± 0.5	30.45 ± 3.8
	Oreganol A	5.37	437	153	ND	98.9 ± 0.6	85.2 ± 3.0
	Oreganol C	5.51	421	153	LOD	8.5 ± 0.3	LOQ
	Rosmarinic acid **	10.57	359	197/161/135	86.4 ± 3.8	245.6 ± 3.5	248 ± 5.3
	Salvianolic acid B **	3.45	717	359/197/179/161/135	LOQ	ND	ND
*Mentha aquatica*	Caffeic acid hexoside	4.382	341	179/135	ND	LOQ	ND
	Diosmin **	7.353	607	299	16.0 ± 0.6	ND	36.4 ± 1.6
	Eriocitrin **	4.743	959	459/287	ND	157.7 ± 7.4	85.4 ± 4.2
	Eriodictyol-7-neohesperoside	3.422	595	459/287	ND	118.7 ± 1.4	40.8 ± 1.2
	Eriodictyol-7-*O*-glucoside	5.963	449	287	ND	20.7 ± 3.6	LOQ
	Hesperidin **	7.975	609	301	LOQ	ND	109.4 ± 0.9
	Chrysoeriol diglucoside	6.173	623	461	LOQ	ND	ND
	Linarin	13.663	591	283	LOQ	ND	LOQ
	Luteolin-7-*O*-glucoside **	14.687	593	286	ND	ND	LOQ
	Rosmarinic acid **	9.873	359	197	157.5 ± 1.7	116.3 ± 7.4	153.0 ± 5.5

* Values (mg/g dry extract) are presented as means ± standard deviation (*n* = 3). Used external standards were: luteolin (used for flavonoid determination, λ = 360 nm); rosmarinic acid (used for the determination of phenolic acids and their derivatives, λ = 320 nm); LOQ—limit of quantification; LOD—limit of detection; ND—not detected; ** confirmed with authentic standards.

**Table 2:** Extract concentrations and total polyphenol content of methanolic extracts from different plant parts of *Mentha aquatica* and *Origanum vulgare*.

Plant Part	Extract Code	Dry Extract Yield (mg/mL )	Total Polyphenols (µg GAE/mL)
*Mentha aquatica* rhizome	MA_R_	1.4	146.05 ± 2.39
*Mentha aquatica* flower	MA_F_	0.7	81.86 ± 1.92
*Mentha aquatica* leaf	MA_L_	3.3	357.86 ± 7.76
*Origanum vulgare* rhizome	OV_R_	2.2	82.15 ± 3.06
*Origanum vulgare* flower	OV_F_	5.8	402.60 ± 4.09
*Origanum vulgare* leaf	OV_L_	6.4	559.01 ± 4.12

Extracts were prepared by macerating 1 g of dried plant material weight in methanol (16 mL), followed by filtration and adjustment to final volumes. The “Dry Extract Yield ” column represents the resulting dry matter content (mg) per mL of liquid extract, while the “Total Polyphenols” column shows the measured phenolic content expressed as µg gallic acid equivalents per mL extract (µg GAE/mL). Values are presented as mean ± SD (*n* = 4).

**Table 3:** IC_50_ (µg/mL), combination index (CI), and dose-reduction index (DRI) values for methanolic extracts of Origanum vulgare (OV) and Mentha aquatica (MA) and their binary mixtures determined by the DPPH and ABTS radical scavenging assays.

					DRI					
	Extracts	IC_50_ (µg/mL) ± SE	CI ± SDA	Description	OV_R_	OV_F_	OV_L_	MA_R_	MA_L_	MA_F_
DPPH	OV_R_ + OV_L_ [2.2:6.4]	5.46 ± 0.25	0.53 ± 0.01	Synergism	13.81	x	2.20	x	x	x
	OV_R_ + OV_F_ [2.2:5.8]	6.27 ± 0.28	0.50 ± 0.01	Synergism	11.17	2.43	x	x	x	x
	OV_F_ + OV_L_ [5.8:6.4]	4.76 ± 0.11	0.48 ± 0.01	Synergism	x	4.88	3.57	x	x	x
	MA_R_ + MA_L_ [1.4:3.3]	27.92 ± 0.74	1.19 ± 0.01	Antagonism	x	x	x	3.39	1.11	x
	MA_R_ + MA_F_ [1.4:0.7]	25.66 ± 1.02	0.90 ± 0.01	Additivity	x	x	x	1.65	x	3.37
	MA_L_ + MA_F_ [3.3:0.7]	23.11 ± 0.62	1.01 ± 0.01	Additivity	x	x	x	x	1.15	7.12
	OV_R_ + MA_R_ [1.1:1.4]	22.71 ± 0.91	0.97 ± 0.01	Additivity	1.93	x	x	2.21	x	x
	OV_F_ + MA_F_ [0.36:1.4]	24.99 ± 0.86	1.15 ± 0.02	Antagonism	x	2.15	x	x	x	1.45
	OV_L_ + MA_L_ [0.4:3.3]	16.41 ± 0.64	0.87 ± 0.01	Synergism	x	x	5.3	x	1.49	X
ABTS	OV_R_ + OV_L_ [2.2:6.4]	1.43 ± 0.13	0.47 ± 0.01	Synergism	20.02	x	2.39	x	x	x
	OV_R_ + OV_F_ [2.2:5.8]	3.99 ± 0.31	0.78 ± 0.03	Synergism	6.69	1.59	x	x	x	x
	OV_F_ + OV_L_ [5.8:6.4]	1.61 ± 0.38	0.50 ± 0.02	Synergism	x	5.99	3.00	x	x	x
	MA_R_ + MA_L_ [1.4:3.3]	50.09 ± 3.47	0.90 ± 0.03	Additivity	x	x	x	4.34	1.49	x
	MA_R_ + MA_F_ [1.4:0.7]	82.26 ± 8.04	1.40 ± 0.05	Antagonism	x	x	x	1.18	x	1.80
	MA_L_ + MA_F_ [3.3:0.7]	53.70 ± 2.80	1.04 ± 0.03	Additivity	x	x	x	x	1.18	5.25
	OV_R_ + MA_R_ [1.1:1.4]	81.64 ± 7.38	5.59 ± 0.32	Antagonism	0.20	x	x	1.42	x	x
	OV_F_ + MA_F_ [0.36:1.4]	15.54 ± 0.84	0.95 ± 0.03	Additivity	x	1.44	x	x	x	4.00
	OV_L_ + MA_L_ [0.4:3.3]	11.19 ± 1.67	0.67 ± 0.02	Synergism	x	x	2.10	x	5.25	x

Abbreviations: OV_R_, OV_F_, and OV_L_ refer to *O. vulgare* rhizome, flower, and leaf extracts, respectively; MA_R_, MA_F_, and MA_L_ correspond to *M. aquatica* rhizome, flower, and leaf extracts. Bracketed values denote relative extract solid ratios (mg/mL) in equivolumetric mixtures.

## Data Availability

All data are presented in this study.

## Abbreviations

The following abbreviations are used in this manuscript:

CI	Combination Index
DPPH	2,2-Diphenyl-1-Picrylhydrazyl
DRI	Dose Reduction Index
IC_50_	Half Maximal Inhibitory Concentration
LC-MS/MS	Liquid Chromatography–Mass Spectrometry
LOD	Limit Of Detection
LOQ	Limit Of Quantification
MA	*Mentha aquatica*
MA_L_	*M. aquatica* Leaves
MA_F_	*M. aquatica* Flowers
MA_R_	*M. aquatica* Rhizomes
[M–H]^−^	Deprotonated Molecular Ion
MS/MS	Tandem Mass Spectrometry
*m*/*z*	Mass-to-Charge Ratio
ND	Not Detected
OV	*Origanum vulgare*
OV_L_	*O. vulgare* Leaves
OV_F_	*O. vulgare* Flowers
OV_R_	*O. vulgare* Rhizomes
RA	Rosmarinic Acid
RT	Retention Time
SDA	Sequential Deletion Analysis
SE	Standard Error
